# Important changes in the timing of influenza epidemics in the WHO European Region over the past 20 years: virological surveillance 1996 to 2016

**DOI:** 10.2807/1560-7917.ES.2018.23.1.17-00302

**Published:** 2018-01-04

**Authors:** Saverio Caini, François Schellevis, Clotilde El-Guerche Séblain, John Paget

**Affiliations:** 1 Netherlands Institute for Health Services Research (NIVEL), Utrecht, The Netherlands; 2 Department of General Practice and Elderly Care Medicine, EMGO Institute for Health and Care research, VU University Medical Center, Amsterdam, The Netherlands; 3 Sanofi Pasteur, Lyon, France

**Keywords:** influenza, Europe, timing of epidemics, epidemic peak, temporal trends, geographical gradient, vaccination campaigns

## Abstract

The global epidemiology of many infectious diseases is changing, but little attention has been paid to whether the timing of seasonal influenza epidemics changed in recent years. This study investigated whether the timing of the peak of influenza epidemics has changed in countries of the World Health Organization (WHO) European Region between 1996 and 2016.  **Methods:** Surveillance data were obtained from the WHO FluNet database. For each country and season (July to June of the next year), the peak was defined as the week with the highest 3-week moving average for reported cases. Linear regression models were used to test for temporal trends in the timing of the epidemic peak in each country and to determine whether this differed geographically.  **Results:** More than 600,000 influenza cases were included from 38 countries of the WHO European Region. The timing of the epidemic peak changed according to a longitudinal gradient, occurring progressively later in Western Europe (e.g. by 2.8 days/season in Spain) and progressively earlier in Eastern Europe (e.g. by 3.5 days/season in the Russian Federation).  **Discussion:** These results were confirmed in several sensitivity analyses. Our findings have implications for influenza control and prevention measures in the WHO European Region, for instance for the implementation of influenza vaccination campaigns.

## Introduction

The global epidemiology of many infectious diseases has changed in recent years; a number of concomitant and mostly anthropogenic factors play a role in this process, including climate change, increased urbanisation, population mobility, deforestation, agricultural intensification and forced displacement of human populations [1]. Most research has focused on malaria and other vector-borne infections [2-4] or on and food- and waterborne diseases [5,6]. The question of whether the timing of seasonal influenza epidemics has changed in recent years has received comparatively little attention. However, influenza seasonality is known to be linked to many of the above factors [7-12], and the temporal characteristics of influenza epidemics may evolve over time as a result of changes in these factors [13,14].

Seasonal influenza epidemics in the northern hemisphere are typically characterised by a short epidemic period of 8 to 12 weeks that varies in intensity during the winter months (November to March) [15] and are associated with substantial morbidity and mortality. Annual vaccination is the most effective measure to reduce the burden of influenza and is most effective when vaccination campaigns coincide optimally with seasonal epidemics. Considering that 2 to 4 weeks may be required to develop an immune response to the vaccine [16] and protection may wane within 6 months [17], the timing of the epidemic peak is an important element that should be considered to optimise the effectiveness of influenza vaccination campaigns.

To assess whether the epidemiology of influenza has changed in the World Health Organization (WHO) European Region (900 million inhabitants), we examined for each country and the Region as a whole whether the timing of epidemic peaks has changed (i.e. occurs earlier or later) between 1996 and 2016.

## Methods

### Data

Influenza virological surveillance data were obtained from the publicly available web-based database FluNet, which is coordinated by the WHO [18]. Information on the weekly number of laboratory-confirmed cases of influenza (overall and by virus type, subtype and lineage) is entered into the FluNet database by the national influenza centres and other influenza reference laboratories of 113 countries participating in the Global Influenza Surveillance and Response System. On 6 November 2016, influenza surveillance data were downloaded for the period between week 27/1996 (starting 1 July 1996) and week 26/2016 (starting 27 June 2016), henceforth referred to as the study period, for all 53 countries in the WHO European Region [19]. We excluded from the dataset the 2009/10 influenza season and influenza A(H1N1)pdm2009 influenza cases reported between April and June 2009 (for all countries), seasons with fewer than 20 weeks of reporting or fewer than 100 influenza cases overall (only for countries where this applied), and countries that had data for fewer than five influenza seasons.

### Definitions

The unit of analysis was the ‘season’, which was defined as the period between 1 July of one year and 30 June of the next year. For each country and season, the peak of the influenza epidemic was defined as the week in which the 3-week moving average of the number of reported influenza cases was highest [20]. Where the peak could not be identified unambiguously, the duration of the moving average was expanded by 2-week increments until the peak could be identified unambiguously. Because a given epidemiological week could start on different days in different years, the week number of the epidemic peak was replaced with the progressive number of the day in a year (1 to 365) corresponding to the Wednesday of that week.

### Statistical analysis

For each country, linear regression models were used to assess the association between the season (independent variable) and the timing of the peak (dependent variables) (Model 1). A beta coefficient > 0 indicated that, with each influenza season, the influenza epidemic peak occurred progressively later; a beta coefficient < 0 indicated the opposite trend.

Linear regression models were then used to assess the association between the geographical coordinates (latitude and longitude) of each country’s centroid [21] (independent variables) and the shift in timing of the peak (Model 2). A beta coefficient > 0 indicated that the shift in timing of the epidemic peak increased moving from east to west (for longitude) or from south to north (for latitude); a beta coefficient < 0 indicated the opposite trend. For all regression models, the 95% confidence interval (CI) for the beta coefficient, the p value, and the coefficient of determination R^2^ were calculated. We did not fit random effect meta-analysis models to obtain a summary beta coefficient for the whole WHO European Region analyses because the country-specific beta coefficients (Model 1) were highly heterogeneous owing to a statistically significant relationship with the country longitude (see Results section).

Influenza epidemics tend to spread according to west-to-east and (less frequently) south-to-north gradients in the WHO European Region [22]. In order to assess whether the duration of influenza activity in the WHO European Region changed in recent years, we defined the duration as the number of days between the epidemic peak in countries in the west and east of the WHO European Region, and used linear regression models to explore whether this varied with time. The countries selected for this analysis were countries that were situated at the western (Portugal and the United Kingdom (UK)) or eastern (Russian Federation) edges of the Region with data available for a large number of seasons (from 2004/05 to 2015/16). A beta coefficient above (below) zero means that the duration of influenza activity in the WHO European Region became progressively longer (shorter) each season during the study period.

All analyses were conducted using Stata version 14 (Stata Corp, College Station, TX). Maps were prepared using Map Chart (http://mapchart.net/). All statistical significance tests were two-sided and p values < 0.05 were considered significant.

### Sensitivity analysis

The impact of very early or late epidemic peaks was assessed by excluding one season at a time from the analyses. The impact of geographical outliers was assessed by first identifying outliers and influential points (i.e. points that significantly influence the output of Model 2) using studentised residuals, Cook’s D and difference in fits (DFFITS) [23,24]) and then by excluding the identified countries from analyses. Finally, to assess the impact of removing countries with limited data, all analyses were repeated excluding countries that had data for less than seven or less than 10 seasons (the basic analysis already excluded countries with less than five seasons, see above).

In order to test the robustness of results, all analyses were repeated using the 3-week moving average of the positive detection rate (defined as the ratio of the number of reported cases over the number of respiratory specimens processed in the same week) instead of the 3-week moving average of the number of reported cases.

## Results

The initial dataset included 819,332 influenza cases from 676 seasons in 49 countries of the WHO European Region; no data were available for Andorra, Cyprus, Monaco and San Marino. After applying the exclusion criteria, the final dataset used for the analysis included 635,046 influenza cases from 400 seasons in 38 countries (Table 1). The median number of seasons per country was 10 and ranged from five (Belarus, Bulgaria, Lithuania, Moldova and Slovakia) to 19 (Finland and Switzerland). The median number of influenza cases reported per season was 601 (interquartile range: 267–1,414).

**Table 1:** Geographical and demographic characteristics and data availability of countries in the included in the analysis, WHO European Region, July 1996–June 2016 (n = 38)

Country	Latitude^a^	Longitude^a^	Population (millions)^b^	Number of seasons with data	Median number of influenza cases per season	Overall number of influenza cases
Austria	47.2 N	13.2 E	8.7	8	850	8,686
Belarus	53.0 N	28.0 E	9.6	5	546	3,869
Belgium	50.5 N	4.0 E	11.3	13	516	7,171
Bulgaria	43.0 N	25.0 E	7.2	5	321	1,526
Croatia	45.1 N	15.3 E	4.5	10	681	7,819
Czech Republic	49.4 N	15.3 E	10.6	11	233	4,215
Denmark	56.0 N	10.0 E	5.6	10	780	14,609
Estonia	59.0 N	26.0 E	1.3	6	618	3,789
Finland	64.0 N	26.0 E	5.5	19	291	7,894
France	46.0 N	2.0 E	66.6	18	2,347	74,566
Georgia	42.0 N	43.3 E	4.9	6	215	1,893
Germany	51.0 N	9.0 E	80.9	17	1,412	25,880
Greece	39.0 N	22.0 E	10.8	10	545	11,206
Hungary	47.0 N	20.0 E	9.9	6	459	2,670
Iceland	65.0 N	18.0 W	0.3	8	148	1,485
Ireland	53.0 N	8.0 W	4.9	9	1,564	10,629
Israel	31.3 N	34.4 E	8	11	763	8,837
Italy	42.5 N	12.5 E	61.9	17	571	17,227
Kazakhstan	48.0 N	68.0 E	18.2	7	598	3,461
Latvia	57.0 N	25.0 E	2	16	551	16,549
Lithuania	56.0 N	24.0 E	2.9	5	633	2,691
Luxembourg	49.4 N	6.1 E	0.6	7	421	2,654
Moldova	47.0 N	29.0 E	3.5	5	214	1,358
The Netherlands	52.3 N	5.4 E	16.9	7	1,033	12,097
Norway	62.0 N	10.0 E	5.2	17	1,127	68,249
Poland	52.0 N	20.0 E	38.6	6	1,088	7,555
Portugal	39.3 N	8.0 W	10.8	17	303	8,956
Romania	46.0 N	25.0 E	21.7	14	335	5,729
Russian Federation	60.0 N	100.0 E	142.4	11	4,629	79,376
Serbia	44.0 N	21.0 E	7.2	6	334	1,809
Slovakia	48.4 N	19.3 E	5.4	5	279	1,657
Slovenia	46.1 N	14.5 E	2	13	425	6,924
Spain	40.0 N	4.0 W	48.1	11	4,697	41,185
Sweden	62.0 N	15.0 E	9.8	15	2,452	57,236
Switzerland	47.0 N	8.0 E	8.1	19	361	20,771
Turkey	39.0 N	35.0 E	79.4	7	1,056	14,725
Ukraine	49.0 N	32.0 E	44.4	6	620	6,586
United Kingdom	54.0 N	2.0 W	64.1	17	1,079	61,507

E: east; N: north; S: south; W: west; WHO: World Health Organization.

Source: WHO FluNet database [18] for July 1996 to June 2016. Data from July 2009 to June 2010 were excluded.

^a^ Latitude and longitude are for the centroid or centre point of the country.

^b^ Most recent estimates [35].

### Timing of the influenza epidemic peak

**Figure 1:**
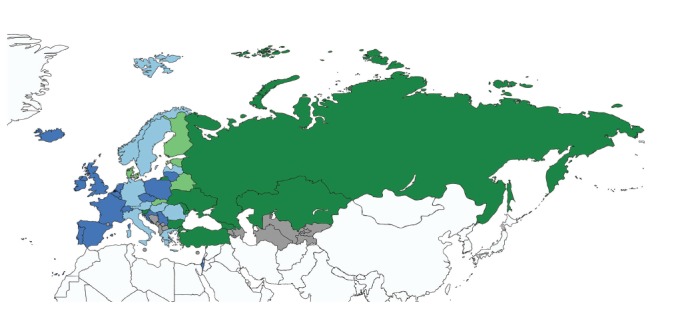
Countries where the peak of reported influenza cases has occurred progressively later or earlier, WHO European Region, July 1996–June 2016 (n = 53)

The peak of the influenza epidemics occurred progressively later during the study period in 25 countries (Figure 1 and Table 2). The trend was statistically significant in Belgium, the Czech Republic, Portugal, Spain and Switzerland. In 13 other European countries, the influenza epidemic occurred progressively earlier, with a statistically significant trend in the Russian Federation and Ukraine.

Dark blue: later by ≥1 day every year; light blue: later by 0–<1 days every year; dark green: earlier by ≥1 day every year; light green: earlier by 0–<1 days every year, averaged over the study period; grey: countries not included in the analysis.

Source: WHO FluNet database [18] for July 1996 to June 2016. Data for July 2009 to June 2010 were excluded.

**Table 2:** Temporal shift of the influenza epidemic peak in countries of the WHO European Region, July 1996–June 2016 (n = 38)

Country	Latitude^a^	Longitude^a^	Number of seasons with data	Temporal shift of the epidemic peak (days/season)			
				beta^b^	95% CI	p value	R2
Austria	47.2 N	13.2 E	8	0.80	−2.19 to 3.79	0.539	6.6%
Belarus	53.0 N	28.0 E	5	−0.15	−21.88 to 21.58	0.984	0.0%
Belgium	50.5 N	4.0 E	13	2.56	0.09 to 5.04	0.044	32.0%
Bulgaria	43.0 N	25.0 E	5	−1.90	−14.67 to 10.87	0.668	7.0%
Croatia	45.1 N	15.3 E	10	1.06	−2.62 to 4.73	0.526	5.2%
Czech Republic	49.4 N	15.3 E	11	2.69	0.36 to 5.03	0.028	43.0%
Denmark	56.0 N	10.0 E	10	−0.34	−4.65 to 3.97	0.861	0.4%
Estonia	59.0 N	26.0 E	6	−0.81	−9.66 to 8.03	0.811	1.6%
Finland	64.0 N	26.0 E	19	−0.14	−2.04 to 1.75	0.874	0.2%
France	46.0 N	2.0 E	18	1.77	−0.08 to 3.61	0.059	20.5%
Georgia	42.0 N	43.3 E	6	−5.03	−13.9 to 3.84	0.191	38.2%
Germany	51.0 N	9.0 E	17	0.95	−0.74 to 2.64	0.248	8.8%
Greece	39.0 N	22.0 E	10	0.76	−2.91 to 4.43	0.645	2.8%
Hungary	47.0 N	20.0 E	6	0.97	−8.02 to 9.97	0.779	2.2%
Iceland	65.0 N	18.0 W	8	1.93	−1.23 to 5.08	0.186	27.1%
Ireland	53.0 N	8.0 W	9	3.70	−2.07 to 9.48	0.173	24.7%
Israel	31.3 N	34.4 E	11	2.78	−0.85 to 6.42	0.117	25.0%
Italy	42.5 N	12.5 E	17	0.82	−0.76 to 2.40	0.288	7.5%
Kazakhstan	48.0 N	68.0 E	7	−2.77	−7.19 to 1.66	0.169	34.0%
Latvia	57.0 N	25.0 E	16	0.93	−1.59 to 3.45	0.443	4.3%
Lithuania	56.0 N	24.0 E	5	4.86	−15.01 to 24.74	0.493	16.8%
Luxembourg	49.4 N	6.1 E	7	3.15	−1.70 to 7.99	0.156	35.8%
Moldova	47.0 N	29.0 E	5	−1.66	−6.25 to 2.93	0.333	30.7%
The Netherlands	52.3 N	5.4 E	7	5.01	−3.99 to 14.01	0.212	29.0%
Norway	62.0 N	10.0 E	17	0.38	−2.22 to 2.99	0.758	0.7%
Poland	52.0 N	20.0 E	6	5.41	−3.30 to 14.12	0.160	42.7%
Portugal	39.3 N	8.0 W	17	3.06	0.61 to 5.51	0.018	32.1%
Romania	46.0 N	25.0 E	14	0.52	−2.69 to 3.74	0.729	1.0%
Russian Federation	60.0 N	100.0 E	11	−3.48	−6.58 to -0.38	0.032	41.8%
Serbia	44.0 N	21.0 E	6	8.17	−3.15 to 19.50	0.116	50.1%
Slovakia	48.4 N	19.3 E	5	−0.08	−0.62 to 0.46	0.654	7.6%
Slovenia	46.1 N	14.5 E	13	−1.67	−4.48 to 1.15	0.219	13.4%
Spain	40.0 N	4.0 W	11	2.78	0.30 to 5.26	0.032	41.7%
Sweden	62.0 N	15.0 E	15	0.74	−1.79 to 3.26	0.540	3.0%
Switzerland	47.0 N	8.0 E	19	2.12	0.26 to 3.98	0.028	25.4%
Turkey	39.0 N	35.0 E	7	−1.85	−15.66 to 11.96	0.745	2.3%
Ukraine	49.0 N	32.0 E	6	−9.63	−17.68 to -1.58	0.029	73.4%
United Kingdom	54.0 N	2.0 W	17	2.26	−0.80 to 5.32	0.136	14.2%

CI: confidence interval; E: east; N: north; S: south; W: west; WHO: World Health Organization.

Source: WHO FluNet database [18] for July 1996 to June 2016. Data from July 2009 to June 2010 were excluded.

^a^ Latitude and longitude are for the centroid or centre point of the country.

^b^ Obtained from country-specific linear regression models using the season as independent variable and of the peak of the influenza epidemic (day of the year; see text for definition) as dependent variable. A value of the beta coefficient above (below) zero means that the peak occurred progressively later (earlier) each season during the study period.

Linear regression analysis indicated a statistically significant longitudinal gradient for the temporal shift of the epidemic peak in the WHO European Region (beta = 0.077; 95% CI: 0.034–0.121; p = 0.001; R^2^ = 26.6%) (Table 3). 

**Table 3:** Relationship between the temporal shift of the influenza epidemic peak and a country’s longitude, and results of sensitivity analysis, WHO European Region, July 1996–June 2016 (n = 38)

Dependent variable	Temporal shift of the influenza epidemic peak (days/season)			
Independent variable	Country longitude^a^			
Model output	beta^b^	95% CI	p value	R^2^
All countries included (n = 38)	0.077	0.034 to 0.121	0.001	26.6%
One season removed at a time (range)	0.056	0.018 to 0.095	0.005	24.0%
	0.091	0.037 to 0.145	0.002	27.0%
Countries with ≥7 seasons of data (n = 27)	0.062	0.039 to 0.085	< 0.001	53.5%
Countries with ≥10 seasons of data (n = 20)	0.052	0.026 to 0.077	< 0.001	50.5%
Outliers	Serbia, Ukraine			
All countries except outliers	0.071	0.040 to 0.100	< 0.001	40.3%
Influential points	Serbia, Ukraine, Georgia, Russian Federation			
All countries except influential points	0.063	0.035 to 0.092	< 0.001	38.5%

CI: confidence interval; WHO: World Health Organization.

Source: WHO FluNet database [18] for July 1996 to June 2016. Data from July 2009 to June 2010 were excluded.

^a^ Latitude and longitude are for the centroid or (if not available) the centre point of each country.

^b^ A beta coefficient above (below) zero means that the temporal shift of the influenza peak was greater (smaller) by moving from east to west.

**Figure 2:**
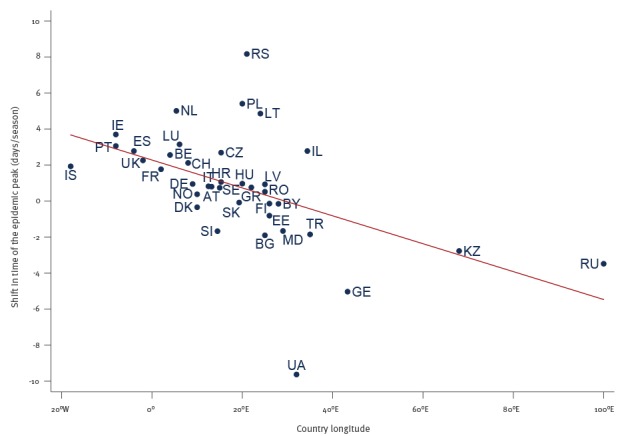
Association between a country’s longitude and the country-specific temporal shift of the influenza epidemic peak, WHO European Region, July 1996–June 2016 (n = 38)

The peak of the epidemic occurred on average 2.28 days later every season at longitude 0° (95% CI: 1.07–3.49; p = 0.001), was stable over time at longitude ca 30° E, and occurred earlier every season at more eastern longitudes (by 2.37 days at 60° E) (Figure 2).

WHO: World Health Organization.

Source: WHO FluNet database [18] for July 1996 to June 2016. Data for July 2009 to June 2010 were excluded.

Country codes (ISO 3166–1 alpha-2): AT: Austria; BE: Belgium; BG: Bulgaria; BY: Belarus; CH: Switzerland; CZ: Czech Republic; DE: Germany; DK: Denmark; EE: Estonia; ES: Spain; FI: Finland; FR: France; GE: Georgia; GR: Greece; HR: Croatia; HU: Hungary; IE: Ireland; IL: Israel; IS: Iceland; IT: Italy; KZ: Kazakhstan; LT: Lithuania; LU: Luxembourg; LV: Latvia; MD: Moldova; NL: the Netherlands; NO: Norway; PL: Poland; PT: Portugal; RO: Romania; RS: Serbia; RU: Russian Federation; SE: Sweden; SI: Slovenia; SK: Slovakia; TR: Turkey; UA: Ukraine; UK: United Kingdom.

### Duration of influenza activity in the WHO European region 

The distance in time between the influenza epidemic peak in Portugal and in the Russian Federation decreased by an average 4.42 days per season during the study period (95% CI: 0.49–9.33; p = 0.072; R^2^ = 31.6%). In the comparison between the UK and the Russian Federation, the decrease was 5.99 days per season (95% CI: 0.41–11.56; p = 0.038; R^2^ = 39.6%).

### Sensitivity analyses 

The analysis using the positive detection rate instead of the reported number of laboratory-confirmed influenza cases in Model 1 and Model 2 yielded similar results. The existence of a longitudinal gradient was confirmed using this alternative approach (beta = 0.093; 95% CI: 0.023–0.163; p = 0.010), although a smaller proportion of the variability between countries was explained by the country longitude (R^2^ = 16.9%). Using this alternative approach, the annual delay of the epidemic peak was 2.93 days at longitude 0° (95% CI: 1.03–4.92; p = 0.004) and was null at longitude ca 30° E.

The ‘leave-one-out’ sensitivity analysis confirmed the presence of a longitudinal gradient. The beta coefficient varied between 0.056 (95% CI: 0.018–0.095; p = 0.005; R^2^ = 22.4%) when the 2015/16 season was excluded and 0.091 (95% CI: 0.037–0.145; p = 0.002; R^2^ = 27.0%) when the 2010/11 season was excluded (Table 3). Serbia and Ukraine were identified as outliers and as influential points, and Georgia and the Russian Federation were identified as influential points. Results were similar when these countries and when countries with limited data were excluded (Table 3).

## Discussion

This study showed that a key characteristic of seasonal influenza epidemics – the timing of the peak – has changed in the WHO European Region between 1996 and 2016. Unexpectedly, however, the timing of the peak did not change uniformly across the Region, but instead according to a longitudinal gradient, with influenza epidemics tending to peak progressively later in Western European countries and progressively earlier in Eastern European countries. These results were confirmed in several sensitivity analyses.

Our findings have implications for influenza control and prevention in countries across the WHO European Region. The implementation of vaccination campaigns should be synchronised with the timing of influenza epidemics, considering that the optimal immune response to vaccination may take 2 to 4 weeks to develop [16] and may decline substantially within 6 months [17]. Accordingly, failure to consider a systematic shift in the timing of epidemics (i.e. the timing of their onset, peak and end) may gradually reduce the effectiveness of influenza vaccination programmes. Influenza vaccination campaigns may need to be planned later in Western Europe and earlier in Eastern Europe, especially if the observed trends persist in the coming years. Importantly, we observed exceptions to these general patterns, and each country needs to carefully assess their situation at a national level, i.e. verify that the current timing of vaccination campaigns is still optimal. For example, the peak in Denmark, which is considered to be a country in Western Europe, has remained fairly stable over the years, and this would suggest that the timing of the vaccination campaign may not need to be modified.

One important consequence of these changes is that the overall duration of influenza activity in the WHO European Region (as a whole) has shortened over the past twenty years, with the average interval between peak influenza activity in western and eastern countries declining from nearly 2 months in 2004/05 (with influenza activity first peaking in the west and then in the east) to less than 3 weeks in 2015/16 (with peaks typically occurring in February and March in most countries [25]). Our findings are particularly relevant for influenza vaccination programmes, as they suggest that the timing of epidemics and, therefore, the optimal time of vaccination has become better aligned across the whole WHO European Region, which in turn allows more coordinated and efficient management of surveillance and prevention efforts.

Changes in national surveillance systems may lead to changes in some metrics of influenza epidemics, such as their duration (e.g. influenza surveillance limited to the period ‘week 40 to week 20’ vs year-round surveillance) and intensity (e.g. following changes in the definitions for influenza-like illness and acute respiratory syndrome). However, the timing of the epidemic peak is less sensitive to how an influenza surveillance system is structured, and we are confident that our findings are not an artefact but the description of an actual phenomenon. Although we found that the timing of the peak of seasonal influenza epidemics in Europe is changing, we did not investigate the possible causes of this change. As mentioned in the introduction, the temporal characteristics (e.g. timing and synchrony between countries) of influenza epidemics are influenced by several factors, including patterns of population mobility such as air travel and commuting [7,26] (especially in countries in Eastern European countries) and climatic and meteorological parameters (such as humidity, temperature and rainfalls) [8-10,12]. For instance, Towers et al. noted a systematic change in the timing of seasonal influenza epidemics in the United States from 1997 to 2013, with warm winters that tended to be followed by severe epidemics with early onset and peak in the following year [27]. However, it is unclear why there is an opposite trend in the timing of influenza epidemic peaks in Western and Eastern Europe, as the above factors are unlikely to have affected Western and Eastern Europe in different ways.

Our findings raise many questions and call for a number of follow-up investigations. Integrating climatic and meteorological time series with influenza surveillance data would help confirm the hypothesis that the changes in the timing of influenza epidemics are due to climate change or develop alternative explanations. The analysis performed here could be extended to earlier seasons to assess when the temporal trends started, to other areas of the world to determine whether similar changes in timing are taking place elsewhere (e.g. North America), and to other aspects of influenza epidemiology to look for other changes (for instance the duration of epidemics). Findings from these additional investigations would help predict future scenarios for influenza epidemiology and would help health authorities take appropriate measures to mitigate the public health consequences of seasonal epidemics. Furthermore, this research approach could be expanded to include other viral respiratory infections such as respiratory syncytial virus [28,29] and other seasonal illnesses such as gastrointestinal infections [30] or even non-infectious diseases like asthma [31,32].

The results of this study are strengthened by the availability of influenza surveillance data for a large number of countries and for an extended period, by the use of straightforward statistical methods and the robustness of results across a range of sensitivity analyses. However, the results should be considered in the light of certain limitations. Although influenza surveillance capacity has improved globally since the 2009 pandemic [33], differences in influenza data collection and reporting remain between countries [34] and for some countries, low data quality may have been an issue. Also, the lack of data stratified by region for the Russian Federation prevented us from assessing whether the longitudinal gradient for the temporal shift of the epidemic peak extends to its Pacific coast or attenuates (or inverts) at some longitude. Furthermore, data were available for only one country in the Caucasus (Georgia) and central Asia (Kazakhstan), which limits our ability to extrapolate the results to these areas. Finally, data were available for only a few (five or six) influenza seasons for 11 countries of the WHO European Region, which may have caused instability in the analysis (i.e. coefficients in Model 1), although the presence of geographical gradients was confirmed in all sensitivity analyses.

## Conclusion

We found that the timing of the peak of influenza epidemics has changed in countries of the WHO European Region between 1996 and 2016. The main drivers behind this phenomenon remain to be clarified, but how the changes might affect influenza prevention and control efforts in Europe demands further attention.
